# Ultrasonographic images of spina bifida before obstetric anesthesia: a case series

**DOI:** 10.1186/s12871-023-02101-4

**Published:** 2023-04-24

**Authors:** Mayuko Doi, Yasuyoshi Sakurai, Daisuke Sakamaki, Soichiro Tanaka, Nobuyuki Katori, Shoichi Uezono

**Affiliations:** Department of Anesthesiology, Jikei University School of Medicine, 3-25-8 Nishishinbashi, Minato-Ku, Tokyo, 105-8461 Japan

**Keywords:** Ultrasound, Obstetric anesthesia, Spina bifida

## Abstract

**Background:**

Spina bifida is a relatively common congenital malformation. As the functional prognosis of patients with spina bifida has improved over time, the number of cases resulting in pregnancy and delivery has increased. Lumbar ultrasonography has become a standard and helpful technique before neuraxial anesthesia. We believe that it might be valuable if we use lumbar ultrasonography to evaluate pregnant women with spina bifida before obstetric anesthesia.

**Case presentation:**

We performed lumbar ultrasonography to evaluate four pregnant women with spina bifida. Patient 1 had no history of surgery. Lumbar radiography before pregnancy showed a bone defect from L5 to the sacrum as a result of incomplete fusion. Magnetic resonance imaging showed a spinal lipoma and a bone defect of the sacrum. Lumbar ultrasonography showed similar findings. We performed general anesthesia for emergency cesarean delivery. Patient 2 underwent surgical repair immediately after birth. Lumbar ultrasonography showed the same bone defect as well as a lipoma beyond the bone defect. We performed general anesthesia for cesarean delivery. Patient 3 had vesicorectal disorders but no prior surgery. Lumbar radiography before pregnancy showed congenital abnormalities such as incomplete fusion, scoliosis, rotation, and a notably small sacrum. Lumbar ultrasonography showed the same bone defect. We performed general anesthesia for cesarean section with no complications. Patient 4 complained of lumbago a few years after her first delivery and received a diagnosis of spina bifida occulta by lumbar radiography, with the incomplete fusion of only the 5th vertebra. Lumbar ultrasonography indicated the same abnormalities. We placed an epidural catheter to avoid the bone abnormality and achieved epidural labor analgesia with no complications.

**Conclusions:**

Lumbar ultrasonography shows anatomic structures easily, safely, and consistently, without X-ray exposure or the need for more expensive modalities. It is a helpful technique to explore anatomic structures potentially complicated by spina bifida before anesthetic procedures.

**Supplementary Information:**

The online version contains supplementary material available at 10.1186/s12871-023-02101-4.

## Background

Spina bifida is a relatively common congenital malformation, with an incidence of 0.26 to 2.9 cases per 1 000 births, and has a broad spectrum of anatomic variations, with or without neurologic dysfunctions [1]. Some patients are latent and classified as spina bifida occulta, without subcutaneous stigmata. Severe spinal anomalies, including spina bifida, can lead to tethered cord syndrome, in which the spinal cone is pulled caudally by lipomas, abnormal soft tissues within the sacrum, or thick filum terminale, which may develop lower extremity muscle weakness, sensory abnormalities, and bladder-rectal disturbances [1]. Some patients involve moderate to severe neurologic symptoms and can require repeated surgery after birth [1, 2]. Spinal abnormalities can make neuraxial anesthesia procedures difficult and result in increased neurologic complications. This syndrome also complicates the management of pregnancy and delivery and increases the need for multidisciplinary team care. Anesthesiologists are also increasingly encountering pregnant women with spina bifida, owing to continuing improvements in functional prognoses [1, 2]. Neuraxial anesthesia for labor analgesia or cesarean section (CS) requires detailed visual information on anatomic structures that might affect the procedure.

Preprocedural imaging of the lumbar spine by computed tomography (CT) or magnetic resonance imaging (MRI) can be crucial for determining the feasibility of neuraxial anesthesia, but there are risks [3, 4], and these modalities can be expensive. Lumbar ultrasonography (US) can be used easily, safely, and consistently to facilitate neuraxial procedures for pregnant women [3, 4]. We present four cases of pregnant women with spina bifida who underwent lumbar US before obstetric analgesia or anesthesia.

## Case presentation

We performed ultrasonographic examinations of the lumbar spine using a convex transducer (Xario 100/S-Edition; Canon Medical Systems Corp, Tokyo, Japan) before obstetric analgesia or anesthesia.

### Case 1

**Fig. 1:**
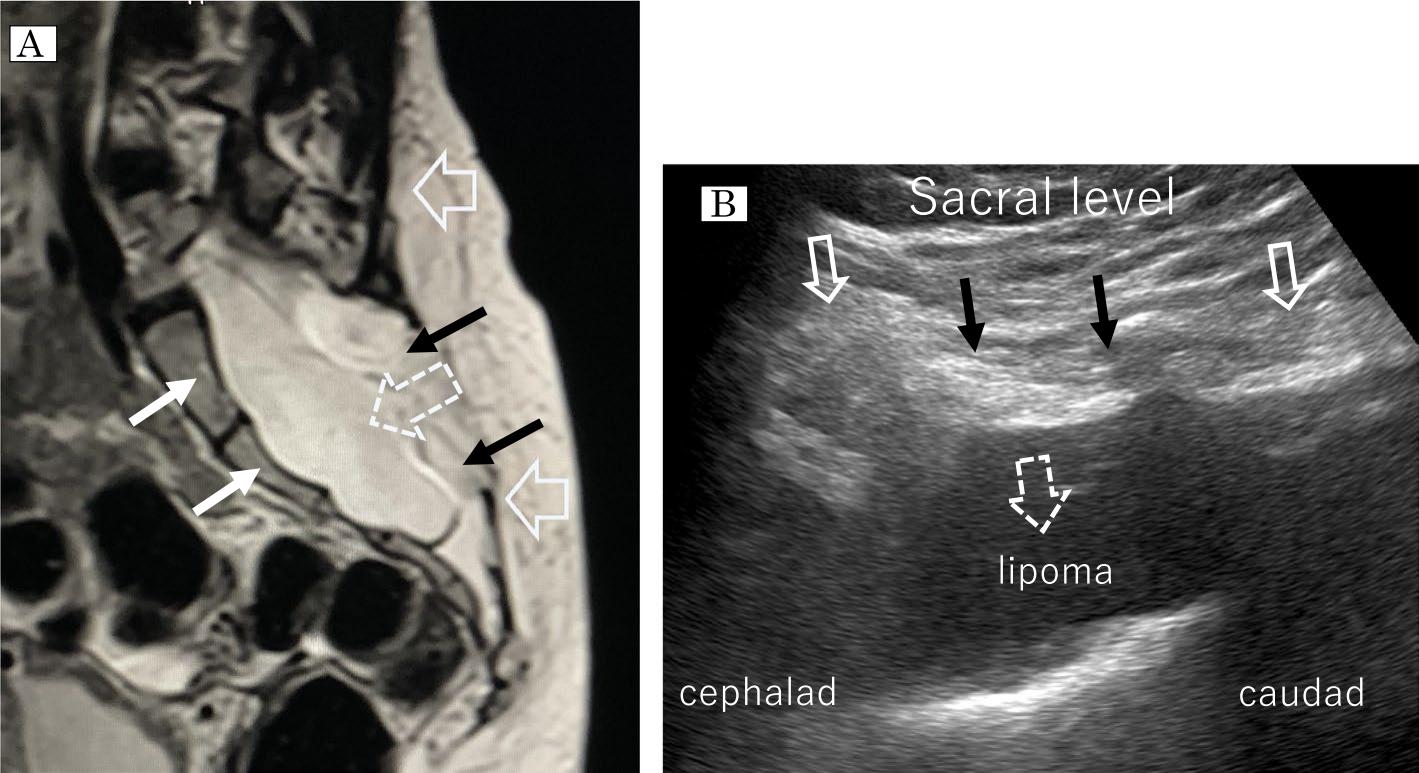
**A** T2- weighted -magnetic resonance image of sagittal view from the lower lumbar spine to the sacrum. Solid arrows show the defect of the normal bony structures from the L5 level to the sacrum. The dashed arrow shows a spinal lipoma at the level of the sacrum. Two black arrows show the soft tissue continuity from the skin to the lipoma. Two white arrows show the segmented frontal sacrum. **B** Ultrasonographic image of sagittal view from the lower lumbar spine to the sacrum. Solid arrows show the defect of the normal bony structures of the sacrum. The dashed arrow shows a spinal lipoma at the level of the sacrum. Two black arrows show the soft tissue continuity from the skin to the lipoma

A 32-year-old woman (height 164 cm; weight 58 kg; gravida 5, para 0) had left lower-limb paralysis and neurogenic bladder but no history of surgery. Lumbar radiography before pregnancy showed a bone defect from L5 to the sacrum, owing to incomplete fusion. MRI before pregnancy showed similar findings, along with a spinal lipoma (Fig. 1A). Lumbar US also showed the spinal lipoma, and a noticeably short distance between the dorsal and ventral dura mater (Fig. 1B). At 35 weeks of gestation, she presented in preterm labor after experiencing unexpected genital bleeding, and an emergency CS was scheduled. We performed general anesthesia in combination with a rectus abdominal sheath block for emergency CS rather than neuraxial anesthesia. The surgery was completed uneventfully.

### Case 2

**Fig. 2:**
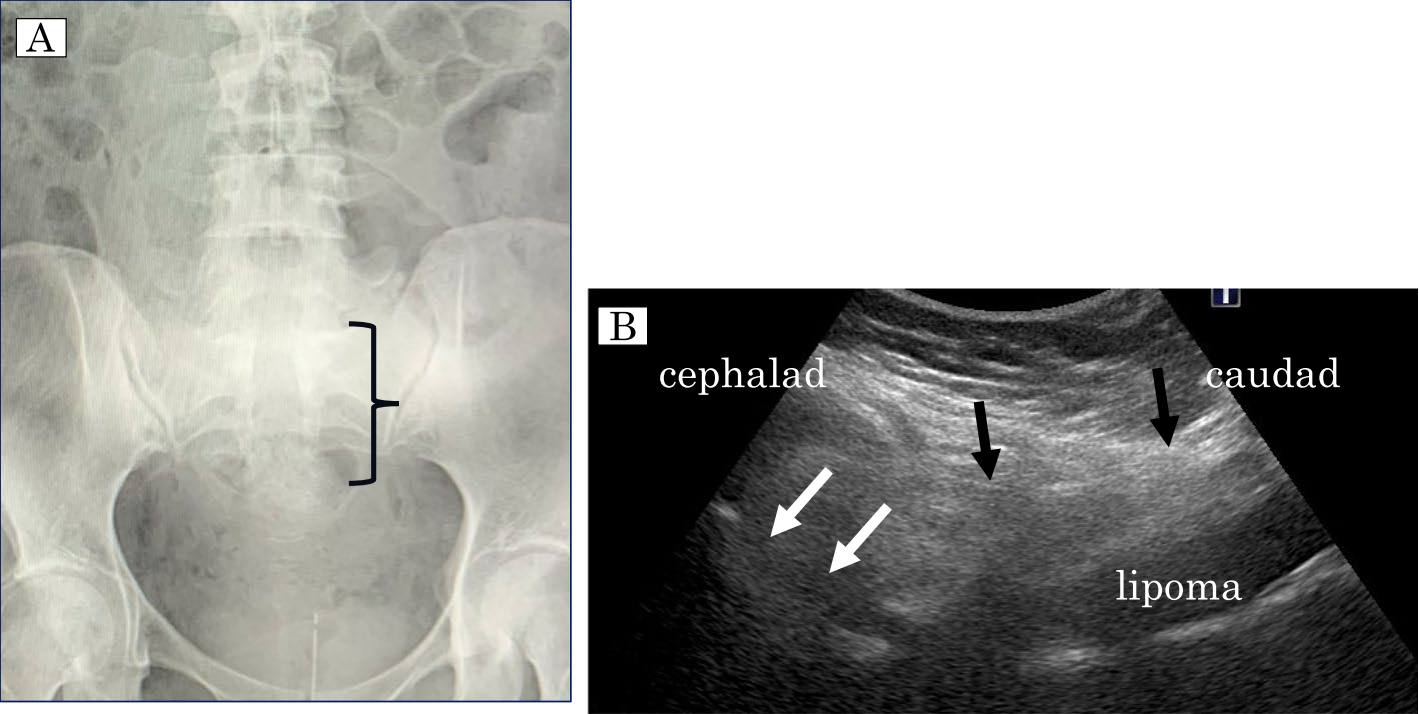
**A** Radiographical image of the lumbar spine on AP view. Large bone defects from L5 to the sacrum are indicated. **B** Ultrasonographic image of sagittal view from lower lumbar spine to sacrum. Large bone defects and lipoma beyond the bone defects are shown. The spinous process of the lower lumbar spine is hypoplastic and the vertebra fusion is incomplete. Two black arrows show the soft tissue continuity from the skin to the lipoma. Two white arrows show the cessation of tissue continuity from the lipoma to the cephalad soft tissue

A 27-year-old woman (height 166 cm; weight 85 kg; gravida 1, para 0) underwent surgical repair for spina bifida immediately after birth. She had dysuria and needed self-guided urination. Lumbar radiography before pregnancy showed bone defects from L5 to the sacrum (Fig. 2A). We performed lumbar US, which showed a lipoma beyond the bone defect (Fig. 2B). A CS was performed under general anesthesia, and there were not any complications.

### Case 3

**Fig. 3:**
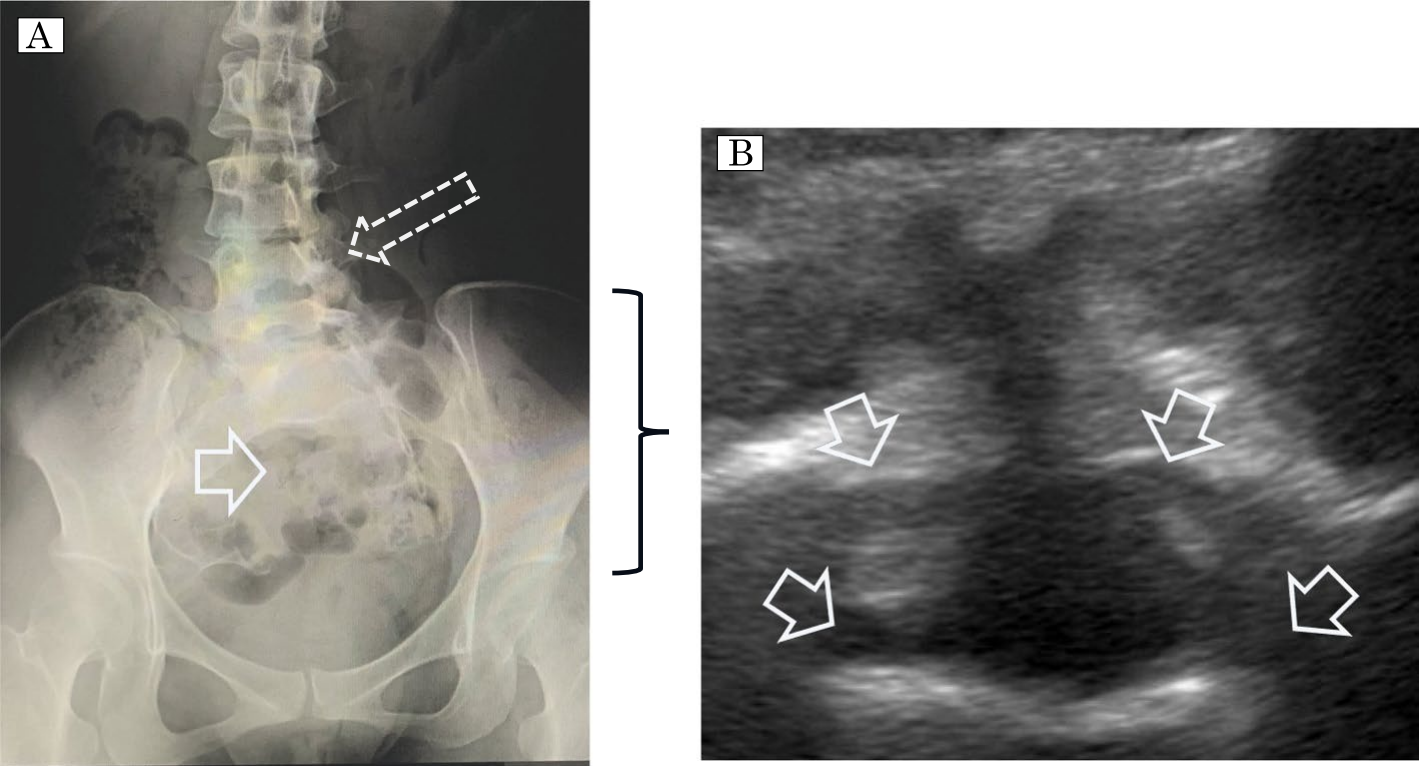
**A** Radiographical image of the lumbar spine on AP view. A solid arrow shows incomplete fusion and a bone defect. The dashed arrow shows scoliosis and rotation from the lower lumbar vertebrae to the sacrum. **B** Ultrasonographic image of axial view at the level of L5. Incomplete fusion of the vertebra at L5 is indicated. Solid arrows showed the incomplete fusion of the vertebra at L5, hypoplasia in the spinous process, and hypoplasia in the vertebral arch of the lumbar spine, similar to the radiographic findings

A 29-year-old woman (height 153 cm; weight 52 kg; gravida 1, para 0) had vesicorectal disorders but no prior surgery. Lumbar radiography before pregnancy showed incomplete fusion and scoliosis and rotation from the lower lumbar vertebrae to the sacrum, with a notably small sacrum (Fig. 3A). Lumbar US showed the bone defect indicated by the radiographic findings (Fig. 3B). We avoided neuraxial blockade and performed general anesthesia for CS, with no complications.

### Case 4

**Fig. 4:**
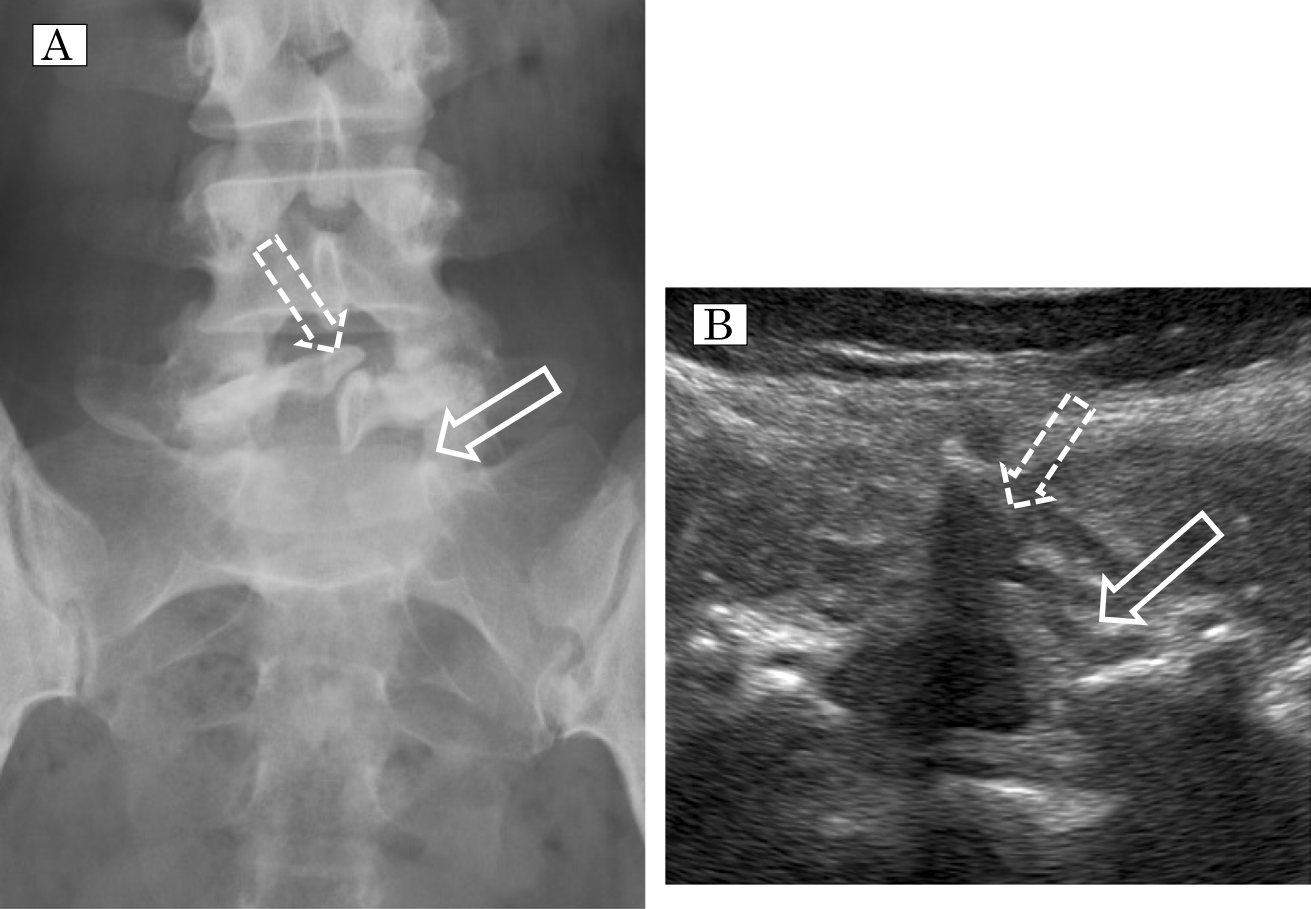
**A** Radiographical image of the lumbar spine on AP view. The solid arrow shows the partial defect of the vertebral arch of at the L5 level. The dashed arrow shows incomplete fusion of the spinous process at L5; higher lumbar levels are unaffected. **B** Ultrasonographic image of axial view at the level of L4/5. The solid arrow shows incomplete fusion of the vertebral arch at L5. The dashed arrow shows the incomplete fusion of the spinous process of the L5 vertebra

A 32-year-old woman (height 156 cm; weight, 73 kg; gravida 2, para 1) gave birth to her first child by epidural labor analgesia, with no complications. She complained of lumbago a few years after the first delivery. She received a diagnosis of spina bifida occulta by lumbar radiography, which indicated incomplete fusion of only the 5th vertebra (Fig. 4A), She then visited our hospital for the delivery of her second child. We performed lumbar US and confirmed incomplete fusion of the 5th vertebra alone (Fig. 4B). We placed an epidural catheter at the level of L3/4 to avoid the abnormal bone regions, resulting in epidural labor analgesia with no complications.

## Discussion and conclusions

Performing neuraxial anesthesia in obstetric patients with spina bifida can be challenging because of increased technical difficulty and the risk of neurologic complications. Recent systematic reviews have indicated that the lumbar US can provide anatomic information indicating the depth of the epidural space, the identity of a given intervertebral level, the location of the midline, and interspinous or interlaminar spaces [3, 4]. In addition, lumbar US can help to improve clinical outcomes of neuraxial anesthesia [5–7]. Preprocedural US might also be helpful to identify parturients compromised by spinal surgeries or diseases [8], especially those with spina bifida before undergoing neuraxial techniques to obtain detailed images of anatomic structures or to reveal spina bifida occulta [9]. In the present study, we performed US for four parturients before anesthetic procedures and obtained distinct images. We obtained real-time images of spinal bone abnormalities, such as insufficient fusion and dysplastic vertebral arch or body, as well as soft-tissue lesions including lipoma. These findings might help in decision-making on the anesthetic methods and in the practice of neuraxial procedures.

Successful epidural anesthesia or analgesia consists of catheter placement without complications and local spread of anesthetic to achieve adequate anesthesia or analgesia. A review of 84 obstetric patients with spina bifida who underwent neuraxial anesthesia, including 41 cases of severe spina bifida, indicated a 20% incidence of inadequate analgesia such as suboptimal or excessive block height, asymmetric block, and rapid block regression and indicated accidental dural puncture, intrathecal catheter migration, increased number of attempts, and post-procedural neurological deficit [10]. We found a case report of a parturient with spina bifida who developed leg weakness after receiving epidural anesthesia during delivery [11]. It is also reported that a parturient with undiagnosed spina bifida who underwent an emergent CS under spinal anesthesia developed foot drop immediately after surgery and MRI imaging revealed a low-lying cord with a fatty filum terminale and intramedullary, suggestive of needle damage [12]. Considering these previous reports [13, 14], general anesthesia might be preferable to neuraxial anesthesia.

In the four cases presented here, we performed lumbar US to explore the anatomical structures of the puncture sites because they had been previously diagnosed with spina bifida. In cases, 1 through 3, we chose general anesthesia rather than neuraxial anesthesia because it was difficult to predict the risk of serious complications related to neuraxial anesthesia based on merely the findings of the US and other imaging.

In case 4, we recognized the history of successful epidural labor and the detailed anatomic structures by the US and lumbar radiographic image. We diagnosed her not with spina bifida but with only incomplete fusion of the 5th vertebra alone. Consequently, we decided to place an epidural catheter at the higher level of L3/4 to avoid the anatomically abnormal regions. As the utility of performing US before neuraxial anesthesia becomes more widespread, the possibility of detecting spinal bony abnormalities, including spina bifida, may increase. During identifying the vertebral level of the puncture from the sacrum, we may notice bone defects in the middle of the sacrum or vertebral bone abnormalities, as presented in our images. We suggest that the US examination might facilitate neuraxial procedures, however, it might not always guarantee successful procedures without complications.

The use of CT and MRI are essential for the diagnosis of spina bifida. However, CT could be potentially harmful to the fetus because of radiational exposure and MRI can be expensive. We recommend the use of CT and or MRI whenever they are beneficial over risks and costs. US does not provide as detailed imaging as CT or MRI, but we showed that the US can be an alternative modality that is easy to use safely and provides consistent results. Although we presented only four cases diagnosed before anesthesia, lumbar US could provide crucial information on anatomic structures to determine anesthetic methods and safer procedures.

In conclusion, preprocedural US can be beneficial for obstetric anesthesia to explore the anatomic structures of the lumbar spine in cases of spina bifida. Preprocedural US for parturients with diagnosed or undiagnosed spina bifida can help with decision-making on anesthetic methods and improve safety related to anesthetic procedures.

## Supplementary Information

**Additional file 1.** T2- weighted -magnetic resonance image of transverse view at the level of to the sacrum. Solid arrows show the soft tissue mass which continue from skin to a lipoma. The dashed arrow shows a spinal lipoma at the level of the sacrum.

**Additional file 2.** Radiographical image from the lumbar spine to the scrum on AP view. A black arrow indicates large bone defect of sacrum.

## Data Availability

All data and materials described in the manuscript will be freely available to any scientist wishing to use them for noncommercial purposes. YS should be contacted to request the data.

## Abbreviations

CS: Cesarean Section
CT: Computed Tomography
MRI: Magnetic Resonance Imaging
US: Ultrasonography
