# Supplementary figures and images for: Ultrasonographic images of spina bifida before obstetric anesthesia: a case series
Source: BMC Anesthesiol. 2023 Apr 24;23:134. doi: 10.1186/s12871-023-02101-4 (PMC10123987; doi:10.1186/s12871-023-02101-4)

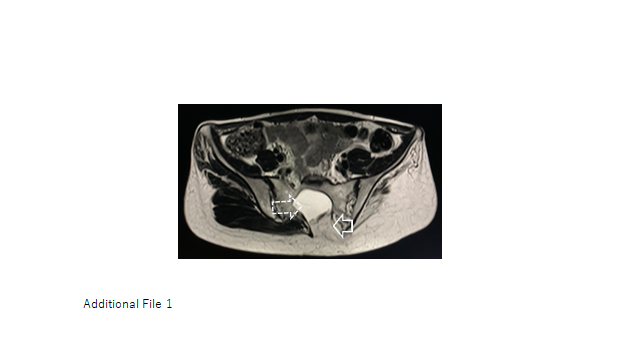

Supplement: Supplementary file 1 — Additional file 1. T2- weighted -magnetic resonance image of transverse view at the level of to the sacrum. Solid arrows show the soft tissue mass which continue from skin to a lipoma. The dashed arrow shows a spinal lipoma at the level of the sacrum. [file 12871_2023_2101_MOESM1_ESM.docx]

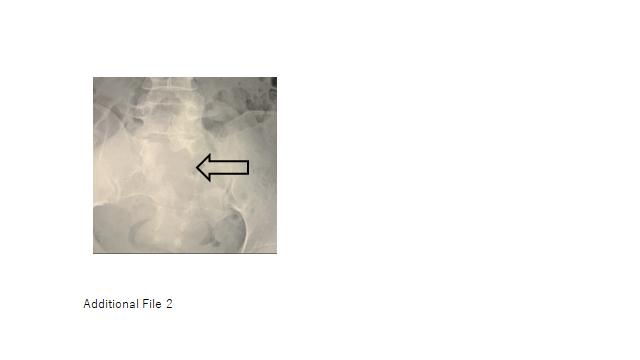

Supplement: Supplementary file 2 — Additional file 2. Radiographical image from the lumbar spine to the scrum on AP view. A black arrow indicates large bone defect of sacrum. [file 12871_2023_2101_MOESM2_ESM.docx]
